# 792 Exoskeleton Robot Using 3-Dimensional Modeling in Burn Patient

**DOI:** 10.1093/jbcr/irac012.342

**Published:** 2022-03-23

**Authors:** Cheong Hoon Seo, So Young Joo

**Affiliations:** Hallym University, Seoul, Seoul-t'ukpyolsi; Hallym University, Yeongdeungpo-gu, Seoul-t'ukpyolsi

## Abstract

**Introduction:**

Hands are the part of the body that are most commonly involved in burns, and the main complications are finger joint contractures and nerve injuries. Hypertrophic scarring cannot be avoided despite early management of acute hand burn injuries, and some patients may need application of an exoskeleton robot to restore hand function. To do this, it is essential to individualize the customization of the robot for each patient. Three-dimensional (3D) technology, which is widely used in the field of implants, anatomical models, and tissue fabrication, makes this goal achievable.

**Methods:**

Therefore, this report is a study on the usefulness of an exoskeleton robot using 3D technology for patients who lost bilateral hand function due to burn injury. Five burn patients with upper limb dysfunction after a flame and chemical burn injury, with resultant impairment of manual physical abilities.

**Results:**

After wearing an exoskeleton robot made using 3D printing technology, the patients could handle objects effectively and satisfactorily.

**Conclusions:**

This innovative approach provided considerable advantages in terms of customization of size and reduction in manufacturing time and costs, thereby showing great potential for use in patients with hand dysfunction after burn injury.

